# Rethinking Environmental Protection: Meeting the Challenges of a Changing World

**DOI:** 10.1289/EHP1465

**Published:** 2017-03-01

**Authors:** Thomas A. Burke, Wayne E. Cascio, Daniel L. Costa, Kacee Deener, Thomas D. Fontaine, Florence A. Fulk, Laura E. Jackson, Wayne R. Munns, Jennifer Orme-Zavaleta, Michael W. Slimak, Valerie G. Zartarian

**Affiliations:** U.S. Environmental Protection Agency, Office of Research and Development, Washington, DC, USA

## Abstract

From climate change to hydraulic fracturing, and from drinking water safety to wildfires, environmental challenges are changing. The United States has made substantial environmental protection progress based on media-specific and single pollutant risk-based frameworks. However, today’s environmental problems are increasingly complex and new scientific approaches and tools are needed to achieve sustainable solutions to protect the environment and public health. In this article, we present examples of today’s environmental challenges and offer an integrated systems approach to address them. We provide a strategic framework and recommendations for advancing the application of science for protecting the environment and public health. We posit that addressing 21st century challenges requires transdisciplinary and systems approaches, new data sources, and stakeholder partnerships. To address these challenges, we outline a process driven by problem formulation with the following steps: *a*) formulate the problem holistically, *b*) gather and synthesize diverse information, *c*) develop and assess options, and *d*) implement sustainable solutions. This process will require new skills and education in systems science, with an emphasis on science translation. A systems-based approach can transcend media- and receptor-specific bounds, integrate diverse information, and recognize the inextricable link between ecology and human health.

## Background

Environmental and public health scientists and decision-makers are addressing new and complex environmental challenges that impact human well-being and ecological health. Energy demands have increased, and sources and approaches to developing energy are changing, raising questions about environmental and human health impacts. Land use patterns are evolving, and land use decisions can impact air, land, and water quality, and consequently, human health. Agriculture and manufacturing are also changing as technology advances. With these changes the focus of environmental protection has expanded beyond local effects and to increasingly recognize the global impacts of human activity on ecological and human health, aptly described as “wicked” problems (Churchman 1967; Rittel and Webber 1973; Stahl 2014).

Wicked problems exist on various spatial scales that unfold over long temporal scales and have possible global implications. They are difficult to define, unstable, and socially complex; have no clear or single solution or end point; and extend beyond the understanding of one discipline or responsibility of one organization (NRC 2012). Because of the complex interdependencies, efforts to solve one aspect of a problem may reveal or create other problems (NRC 2012). Based on these definitions, the environmental pollution problems of today are termed “wicked” problems (NRC 2012).

In this article, we characterize today’s most pressing wicked environmental health problems and, drawing from research conducted by the U.S. Environmental Protection Agency (EPA), Office of Research and Development and other environmental organizations, highlight tools and approaches that can be used to evaluate the many complex dimensions of these problems. Finally, we present a new framework for a systems approach for finding sustainable solutions to these complex problems.

## Discussion

### Today’s Wicked Problems

A number of complex issues have been identified by the scientific community as wicked problems:

*Climate change.* In 2015, 195 countries adopted the first universal climate agreement, noting the need for an effective and progressive response to the urgent threat of climate change (United Nations 2015). An increasing range of global adverse effects from climate change are affecting air quality, water resources, agriculture, and wildlife habitats, as well as basic infrastructure systems such as control of contaminated sites, waste management practices, and the functioning of the built environment (U.S. EPA 2015a). Climate change is altering the distribution and intensity of public health–related stressors (e.g., temperature, vector-borne diseases) and is eroding gains made in controlling air pollution in many urban areas (U.S. EPA 2015a). While some geographic areas may see advantages of a warmer climate (e.g., reductions in death due to extreme cold temperatures), estimates show the net impacts of climate change are likely to be widespread and significant (McCabe and Burke 2016). Without continued emission reductions, the public health and welfare of current and future generations are in jeopardy, and vulnerable citizens, like children, older adults, and people living in poverty, are most at risk (U.S. EPA 2015b).

*Energy.* Choices about future energy sources have far-reaching economic, social, environmental, and public health effects. Energy provides essential support for society. From the household to the industrial setting, it is used to produce and transport goods, move people, and support a productive and growing economy. At the same time, energy production and use affect environmental quality. Oil and gas development, whether conventional or shale oil and gas, pose inherent environmental and public health risks (GAO 2012). Historically, fossil fuel-based energy production and use have affected air quality and the climate, creating emissions of conventional air pollutants and greenhouse gases. As the use of natural gas has expanded, practices such as hydraulic fracturing have raised important questions about potential environmental and public health impacts (GAO 2012). Water quality and quantity are affected because water is needed to produce energy, and the process of producing energy can potentially lead to water contamination. Because energy is central to a strong economy, the quest for cleaner energy sources has driven new technologies to convert sunlight, wind, or geothermal energy into electricity. Likewise, federal regulations related to energy—along with social dimensions such as consumer preference for clean energy—are driving the changing energy landscape. Scientists must be prepared to understand the full scope of these drivers and provide the research and technical knowledge to illuminate the risks and benefits and guide energy policies.

*Land use.* The health and well-being of a community is closely coupled with land use and development. From inner cities to rural farming communities, quality of life and environment can depend upon land use policies. Land use decisions about roads and transportation systems, industrial siting and development, agricultural land use and the provision of community access to healthy and sustainable food, housing, and open space for parks and recreation can all impact human health. The distribution of green space in populated areas is a factor in physical activity, stress, and related physical and mental health issues (Lee and Maheswaran 2010; Lachowycz and Jones 2013). By influencing social interaction and the variety, density, and accessibility of necessities and amenities, decisions regarding land use planning affect well-being through community vibrancy and the autonomy of marginalized populations (Jackson 2003). Land use decisions can drive cascading events that may adversely impact ecological and human health. For example, land use decisions can influence fire risk (Butsic et al. 2015), and wildland fires can alter the landscape, increase erosion, and foster runoff (Morrison and Kolden 2015). Resulting wildland fire smoke, a mixture of gases and fine particles, can cause respiratory illness and aggravate chronic heart and lung diseases (U.S. EPA 2003; Rappold et al. 2011).

*Water quantity and quality.* About 400 billion gallons of water are used each day in the United States, and we face many challenges in maintaining the safety and sustainability of these water resources (U.S. EPA 2015d). For example, emerging chemical contaminants, such as perfluorinated compounds, found nationwide in water supplies, may not be removed by conventional water treatment or addressed by policy or regulatory actions (Sedlak 2016). An aging water system infrastructure has led to an estimated 240,000 water main breaks in the United States annually (ASCE 2013), which can only exacerbate water shortages. The recent water crisis in Flint, Michigan, where lead leached from pipes in older drinking water systems and reached levels that exceeded regulatory limits, also highlighted the importance of proper treatment of source water to prevent such occurrences (Bellinger 2016). Harmful algal blooms (HAB), a natural phenomenon, can be influenced by anthropogenic forces and climate change: and expanding human populations could impact HAB occurrence and public health impacts (Berdalet et al. 2015). Drought is a concern for many communities, and the effects of climate change are expected to increase the frequency, intensity, and duration of droughts in many regions (White House 2016). These examples are just a few of the many challenges threatening the safety and sustainability of the water supply in the United States.

### Connecting the Dots—A Systems Approach to Environmental Protection

Environmental challenges have historically been managed with compartmentalized and pollutant specific, risk-based approaches. Although such approaches were successful in addressing part of the problem in the past, they are ill-suited to solve today’s wicked environmental challenges. Rather, today’s problems call for a systems approach that looks at a problem holistically, includes all the drivers and stressors that affect it and the dimensions that frame it, and integrates information from human health and ecological sciences and the social sciences to formulate sustainable solutions to environmental issues.

**Figure 1:**
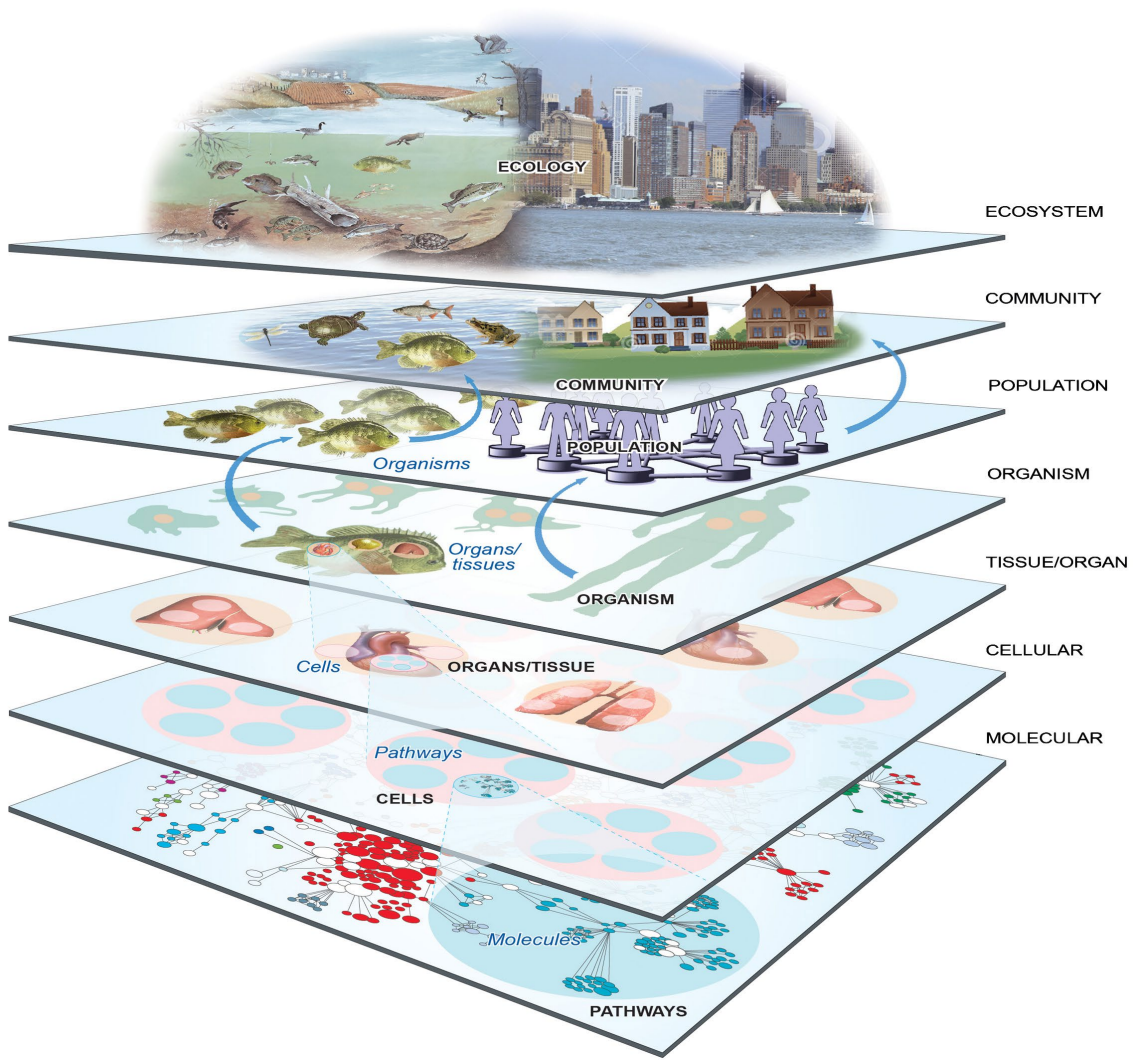
Nested systems from the molecular level to the biosphere.

To understand the links between public health, the environment, and society, the interactions of factors within a complex system must be evaluated in a realistic way, regardless of its size, which can range from the scale of the molecule to that of the biosphere (global ecosystem) (Figure 1). Systems thinking considers the cumulative effects of multiple stressors, evaluates a range of alternatives, analyzes upstream and downstream life-cycle implications, involves a broad range of stakeholders, and uses interdisciplinary scientific approaches (NRC 2012). Systems approaches are not new, and the scientific literature provides many examples (Powers et al. 2012; Briggs 2008; Fiksel 2006). In public health, Guyer (1997) describes a systems process for problem solving that first defines the problem and measures its magnitude, then develops a framework for evaluating the key determinants (biologic, epidemiologic, social, cultural, economic, and political). Contemporary assessments stress the need for systems thinking. For example, a health impact assessment (HIA) uses a systems approach to array data sources and analytic methods and considers input from stakeholders to determine potential effects of a proposed action or decision on the health of a population and the distribution of those effects within the population (NRC 2011). Likewise, a life-cycle assessment uses systems approaches to evaluate a cradle-to-grave process, including all stages of a product’s life from the perspective that they are interdependent (U.S. EPA 2006).

### The Tools of 21st-Century Science and Technology

Concurrent with the changing nature of environmental issues, science and technology are evolving rapidly and offering new tools and methods of analysis needed in taking a systems approach to a problem. For example, modeling real-world scenarios can inform our understanding of interactions within a system, which helps forecast possible intervention outcomes. Computational models, which use and integrate data from many sources to understand and predict system dynamics and impacts of environmental pollutants, have become central to environmental decision-making (NRC 2007). Computational science provides more information than ever before along with the means for analyzing what the information means. The Toxicology Testing in the 21st Century (Tox21), a federal collaborative program that develops high-throughput assays to efficiently test a chemical’s potential to cause adverse health effects (U.S. EPA 2015c), is anticipated to deliver a wealth of information about the potential effects of tens of thousands of chemicals (Attene-Ramos et al. 2013). Computational exposure science, which integrates advances in chemistry, computer science, mathematics, statistics, and social and behavioral sciences with new models and data collection methods, will provide tools to better understand population exposures and link exposures to health outcomes (Egeghy et al. 2016).

Changes in technology have spurred the development of low-cost compact sensors for measuring environmental parameters and indicators of health (Kumar et al. 2015; Murphy et al. 2014; Chan et al. 2012). These sensors can be deployed in multiple locations to monitor pollutant concentrations around a facility or community more accurately than is possible with single stationary monitors (Snyder et al. 2013). Satellite technology can enhance air quality forecasting, emissions estimation, and exposure assessment for human health studies (Hoff and Christopher 2009). The availability of personal computers, mobile phones, and Internet access has revolutionized the communication of information and ideas. Citizen science, which encourages public participation in the scientific process (Kalil and Wilkinson 2015), provides a new way to engage the public in solving problems. Crowdsourcing—an open call for voluntary assistance from a large group of individuals (Kalil and Wilkinson 2015)—can help collect information at large geographic scales and over long periods of time.

These technological advances will yield enormous volumes of complex data, both structured and unstructured, originating from different sources. Big data may revolutionize how we monitor environmental quality and understand how humans interact and respond to the environment (Kays et al. 2015) and how the environment responds to human activity (Dagliati et al. 2015). However, the analysis of and need for access and discoverability of big data presents challenges that include protecting individual interests and privacy, managing enormous volumes of data, identifying the most important types of data, understanding data quality, integrating data into a form to analyze and guide decisions, and making the information publically accessible in forms that can be shared and combined for analysis.

### Moving to the Future

**Figure 2:**
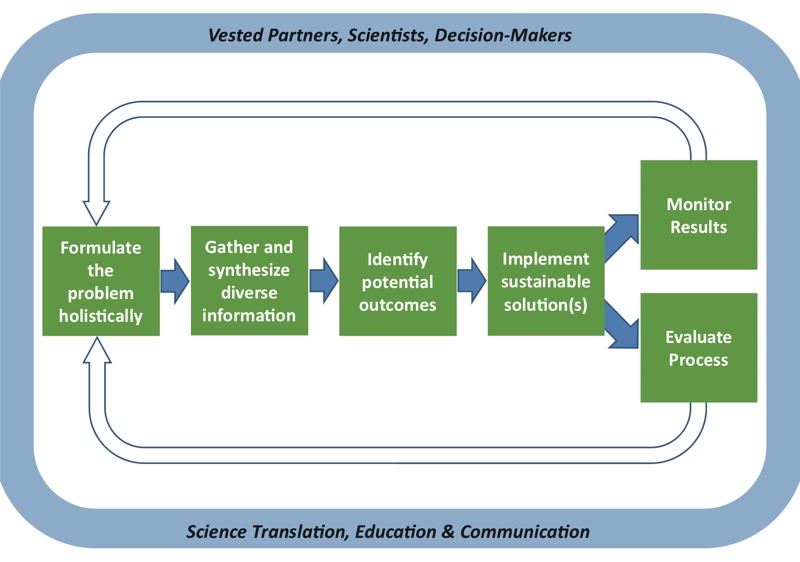
Framework for applying integrated science to protect the environment and public health and well-being.

Moving forward, we need a new comprehensive approach to solve environmental challenges that *a*) begins with strong problem formulation, *b*) relies on systems approaches and tools to integrate different types of data from multiple disciplines, *c*) draws on information generated from new technologies, and *d*) considers novel sources of data, such as citizen science. Evolving from case experiences, tools, and approaches developed over the years, we propose adopting a new framework (Figure 2) for environmental science that uses a systems approach to integrate ecological and human health information to solve environmental challenges. This framework includes the following elements and considers vested partners, communities, scientists, decision makers, and the public, and the need for science translation, education, and communication. Table 1 describes each element, summarizes the approaches, and provides examples of tools designed to facilitate its use.

**Table 1:** Considerations, information sources, tools, and approaches for framework elements.

Considerations and types of information	Example tools and approaches
Step 1 – Formulate the problem holistically
Systems context: social, environmental, economic. Values and goals of vested partners. Spatial and temporal dimensions. Interdependencies, interactions, unintended and cumulative effects. Uncertainties, knowledge gaps. Complexity versus sufficiency.	Conceptual model (Suter 1999). Structured decision-making (Gregory et al. 2012; Yee et al. 2015). Bayesian Belief Network (Rehr et al. 2014). Health Impact Assessment (NRC 2011). Framework for Human Health Risk Assessment to Inform Decision-Making (U.S. EPA 2014b).
Step 2 – Gather and synthesize diverse information
Socioeconomic status; health and cultural resources. Local knowledge, traditions and practices, legacy land usage. Built environment design and level of services. Existing natural and anthropogenic hazards. Beneficial “green” exposures and natural buffers from hazards. Spatial overlays, future trajectories, opportunities, and risks.	C-FERST (Zartarian et al. 2011). Smart Location Database (Ramsey and Bell 2014). EnviroAtlas (Pickard et al. 2015). Eco-Health Relationship Browser (Jackson et al. 2013). Environmental Quality Index (U.S. EPA 2014a).
Step 3 – Develop and assess options
Understand full consequences of potential decisions or policies. Consider stakeholder, community priorities and concerns. Assess benefits, risks, trade-offs, and costs (monetary and nonmonetary) for different scenarios. Estimate distribution of impacts (positive and negative) across vulnerable populations and life stages. Consider population vulnerability versus individual risk. Identify feasible, actionable, near- and long-term actions that mitigate negative impacts/consequences and promote sustainability and resilience.	3VS (Fiksel et al. 2014), HYGEIA (Phillips et al. 2014), DASEES (Yeardley et al. 2011). Structured decision-making (Gregory et al. 2012; Yee et al. 2015). Health Impact Assessment (Gottlieb et al. 2011). Eco-Health Relationship Browser (Jackson et al. 2013). Human health and ecological risk assessment (http://www.epa.gov/risk). Environmental justice analysis (e.g., EJ Screen, EnviroAtlas, C-FERST). Community engagement.
Step 4 – Implement sustainable solution(s)
Select suite of actions to implement preferred solution(s) (e.g., policies, programs, interventions, preventions, etc.) to work toward outcomes. Include short- and long-term actions as appropriate. Communicate science and evidence-based solutions to stakeholders, decision-makers, communities. Ensure transparency and translation. Empower communities/people with knowledge, tools, and data.	Eco-Health Relationship Browser (Jackson et al. 2013). HYGEIA (Phillips et al. 2014). Framework for Human Health Risk Assessment to Inform Decision-Making (U.S. EPA 2014b).
Step 5 – Monitor and evaluate results
Evaluate if approach provided sufficient information to identify, discriminate amongst, and implement solutions. Identify key indicators or data sets to reflect changes in environmental conditions or human health and well-being. Consider unconventional data sources to inform monitoring and evaluation. Pinpoint key questions or information from problem formulation that can inform scientific questions for evaluation. Assess goals and values affected by solution alternatives and determine which can be used to inform end points or indicators for evaluation.	EPA Report on the Environment (U.S. EPA 2015c). EnviroAtlas (Pickard et al. 2015). CDC Environmental Public Health Tracking Network (http://www.cdc.gov/nceh/tracking/). National Aquatic Resource Surveys (including Watershed Integrity) (http://www.epa.gov/national-aquatic-resource-surveys). Citizen science (https://www.epa.gov/sites/production/files/2015-02/documents/citizen-science-fact-sheet.pdf).

*Formulate the problem holistically.* Environmental health problems should be framed within a systems context and should consider ecological, health, social, and economic factors across space and time. Interactions, interdependencies, and cumulative effects are considered, as are the values and goals of vested partners, including the community and the public. By engaging end users early in the process, information and solutions will be more responsive and relevant to their needs. Formulating the problem holistically will improve understanding of potential unanticipated outcomes. Tools and guidance for problem formulation exist. For example, Suter (1993) described the process of creating a conceptual model for ecological risk assessments. This approach can help inform our understanding of system linkages, points of potential intervention, and the information needed to inform policy decisions. Gregory et al. (2012) and Yee et al. (2015) proposed a structured decision-making process, and Bruins et al. (2010) demonstrated the use of problem formulation for addressing complex socio-environmental problems. The U.S. EPA’s “Framework for Human Health Risk Assessment to Inform Decision-Making” (U.S. EPA 2014b) describes the importance of problem formulation and provides information to consider during this process.

*Gather and synthesize diverse information.* Guided by problem formulation, the next step is to identify diverse data and information needed to support the assessment. Economic, social, and environmental information should be considered, including socioeconomic status, health, cultural resources, local knowledge, traditions and practices, and existing conditions of the built and natural environment. For example, a more holistic model based on a systems approach was recently proposed for improving children’s environments and health across developmental life stages (Tulve et al. 2016). Various tools can inform this step. Ideally, they should be discoverable and widely accessible to users in web-based formats. For example, the “Community-Focused Exposure and Risk Screening Tool” (C-FERST; https://www.epa.gov/c-ferst), a community mapping, information access tool, can inform community assessments and decision-making (Zartarian et al. 2011). “EnviroAtlas,” an interactive mapping tool, can be used to explore the benefits people receive from nature (Pickard et al. 2015). The EnviroAtlas Eco-Health Relationship browser (https://www.epa.gov/enviroatlas/enviroatlas-eco-health-relationship-browser) provides information about how health issues are linked to the metrics of ecosystem services—the societal benefits from nature that underpin almost every aspect of human well-being (Jackson et al. 2013; U.S. EPA 2015d). The “Environmental Quality Index” (EQI) provides a metric for overall environmental quality that incorporates air, water, land, the built environment, and sociodemographics (U.S. EPA 2014a).

*Develop and assess options.* This step helps inform understanding of the consequences of potential decisions under consideration. The benefits and risks of options should be assessed and tradeoffs and costs (monetary and nonmonetary) should be examined under different scenarios. The priorities and concerns of the community and stakeholders should be considered. This step also includes estimating the distribution of impacts or consequences (positive and negative) across the population, including at-risk populations such as children, older adults, pregnant and nursing women, and indigenous people, while considering population vulnerability versus individual risk. At this point, feasible near- and long-term actions that mitigate negative impacts and promote sustainability and resiliency are identified. A variety of traditional and newer tools can be applied. For example, human health and ecological risk assessment will add valuable information about the impacts of various stressors. HIA can provide a structure for assembling information and assessing options, as can structured decision-making (Gregory et al. 2012; Yee et al. 2015). A web-based decision analysis framework called “Decision Analysis for a Sustainable Environment, Economy, and Society” (DASEES) can help inform this process (Yeardley et al. 2011). Environmental justice analysis, using mapping tools like C-FERST, EnviroAtlas, and “EJ-SCREEN: Environmental Justice Screening and Mapping Tool” (http://www.epa.gov/ejscreen), can provide valuable information about sensitive populations and population risk.

*Implement sustainable solutions.* Here, the suite of actions to implement solution(s) is selected. Solutions may range from improved infrastructure to interventions to behavioral changes. Implementers may include government agencies, state or local governments, or other stakeholders. These actions might include short- or long-term elements such as installation of a green street to reduce localized flooding combined with development of an area-wide plan for green infrastructure to improve overall water flow in a community. Communicating the scientific basis of solutions to decision makers, communities, and other stakeholders is essential. Ensuring transparency is crucial, as is engaging and empowering communities with knowledge, tools, data, and information.

*Monitor and evaluate results.* This step evaluates whether the approach provided sufficient information to identify, compare, and implement solutions and whether the chosen solution has the desired short- and long-term positive effects. Certain indicators or data sets could be used to reflect changes in environmental conditions or human health and well-being over time. For example, the “EPA Report on the Environment” provides indicators of national trends in air, water, land, human exposure and health, and ecological condition (U.S. EPA 2015c), and the EQI provides a single index of environmental quality that accounts for the multiple domains of the environment that encompass an area where humans interact (Lobdell et al. 2011). The “EnviroAtlas” may be useful for monitoring and evaluating solutions at various spatial scales. Consideration should also be given to whether unconventional data sources—such as citizen science—can inform evaluation.

Environmental protection in today’s world requires recognition of the interconnection of our environmental systems. This framework provides a structure to address today’s complex problems by considering multiple dimensions and a variety of data sources—a systems approach. Similar frameworks exist and have provided the basis for this approach (Reis et al. 2013; Powers et al. 2012; Briggs 2008). However, this framework represents an evolution of what has been proposed and used to date, and it provides a construct through which environmental and public health scientists can conduct future research, both fundamental and translational, to inform tomorrow’s solutions. We acknowledge the tension between using this framework and traditional approaches, including those driven by regulatory statutes and policies. We are not recommending replacement of those policies that have led to measurable progress. Rather, we recommend systems thinking as a path to enrichment of the scientific basis for decision-making to address wicked problems by creating opportunities for new partnerships and enhancing collaboration across traditional media-specific silos.

### Recommendations for Framework Implementation

*Problem formulation as a key step toward integrating science to support systems-based problem solving.* The framework presented here is grounded in strong problem formulation. This step is essential for successfully assessing issues and formulating and evaluating options. The environmental science community should be trained in approaches to problem formulation, and environmental and public health organizations should seek opportunities to incorporate problem formulation in their scientific approaches.
*Integrate additional skill sets into environmental problem solving.* Informing solutions to complex environmental problems requires insight, expertise, and viewpoints from many scientific disciplines, along with policy makers, public officials, and community stakeholders. Traditionally, the fields of ecology, toxicology, and engineering have been predominant in environmental science. To conduct systems-based science, scientific teams will also need to include public health practitioners, earth scientists, economists, behavioral and other social scientists, database managers, programmers, software engineers, planners, physicians, systems analysts/experts, and science communicators.
*Make systems approaches core in the education of future scientists and decision-makers.* Traditional training in environmental science has taken a reductionist approach to focus on specific mechanisms of a stressor and its effect on an ecosystem or human health. However, science students today are increasingly trained to look at the system and embrace cross-disciplinary problem solving. Current and future environmental scientists will need to be trained on systems approaches for conducting science and solving problems. A compilation of systems-based tools and examples of how systems approaches can be applied to inform sustainable solutions will help ensure that environmental scientists are adequately trained.
*Use effective science communication to ensure that decision makers and communities understand and accept the science.* This framework requires scientists to work closely with vested partners and decision makers and ensure the science is translated and communicated throughout the process. As with the division of risk assessment and risk management articulated by NRC (1983), scientists typically do not choose a solution or make a policy or risk management decision. Therefore, it is critical that the science is communicated clearly and that decision-makers and vested partners are educated about the science. Science communication experts will be needed, and scientists will need to be better trained in effective communication.

## Conclusions

U.S. EPA authorities have successfully managed gross pollution problems using command and control media-specific approaches. The health of our rivers has improved, the vast majority of Americans have access to safe and clean drinking water, exposure to many toxic pollutants and pesticides has been reduced, and nationwide air quality has improved significantly for many air pollutants (U.S. EPA 2012). However, today’s environmental problems are daunting. Their dimensions go well beyond the traditional risk assessment and risk management paradigm that has been the basis of environmental protection over the past several decades. It is time to embrace a new way of thinking. From safe drinking water to energy choices and pest management, to urban design, systems approaches can help inform sustainable solutions that ensure environmental and public health protection. In times of emergency response, systems approaches will help us understand the multiple dimensions of the situation, how the environment and human health are impacted, and how various solutions may address the issue or potentially cause unanticipated consequences. Wicked problems require thoughtful synthesis of science and decision-making. The framework proposed here provides a much-needed structure, grounded in strong problem formulation, to build upon our progress and strengthen environmental and public health protection for the future.
